# The cerebral mechanism underlying the acupoints with specific effect for gallbladder stone disease: protocol for a randomized controlled task-fMRI trial

**DOI:** 10.1186/s13063-021-05356-9

**Published:** 2021-06-14

**Authors:** Ning Sun, Yuan-Fang Zhou, Jie Zhou, Wen-Wei Zuo, Xiang-Yin Ye, Xiao-Dong Deng, Zheng-Jie Li, Shi-Rui Cheng, Yu-Zhu Qu, Jun Zhou, Rui-Rui Sun, Fan-Rong Liang

**Affiliations:** 1 Acupuncture and Tuina School/The 3rd Teaching Hospital, Chengdu University of Traditional Chinese Medicine, Clinical Research Center for Acupuncture and Moxibustion in Sichuan province, 37 Shierqiao Road, Chengdu, 610075 Sichuan China; 2 The Third Clinical Medical College, Zhejiang Chinese Medical University, Hangzhou, 310000 Zhejiang China; 3 The First Affiliated Hospital of Chengdu University of Traditional Chinese Medicine, Chengdu, 610073 Sichuan China

**Keywords:** Acupuncture, Gallbladder stone disease, Central mechanism, Functional magnetic resonance imaging, Task fMRI, Clinical trial, Protocol

## Abstract

**Background:**

As it has been recorded in ancient Chinese classics, Yanglingquan (GB34) and Dannangxue (EX-LE6) are two important acupoints that can regulate the function of the gallbladder. Acupuncture at these two acupoints is considered particularly effective for gallbladder disease treatment, especially for alleviating gallbladder stone disease (GSD) symptoms that can be aggravated after intaking high-fat food. However, the superior effect between the two acupoints still needs to be further explored, as well as the underlying central mechanism has never been investigated to date.

**Methods and design:**

Ninety participants diagnosed with GSD will be randomly divided into group A (acupuncture at GB34), group B (acupuncture at EX-LE6), and group C (acupuncture at non-acupoint) in a ratio of 1:1:1. All of them will receive a 30-min acupuncture treatment with fatty-food cues being presented before and after acupuncture. During the task, participants will be scanned by MRI and required to rate their desire for high-/low-fat food with an 11-point Likert scale. Additionally, the participants’ pain/discomfort sensation will be evaluated using the Numeric Rating Scale (NRS) at four timepoints, including before the 1st task fMRI scan, before and after acupuncture, and after the 2nd task fMRI scan. For both behavior and fMRI data, the ANOVA analysis will be conducted among three groups to testify the immediate effect of GB34 and EX-LE6. The post hoc t-test will be employed to further explore the superiority between acupuncture with GB34 and EX-LE6. Furthermore, correlation analyses will be conducted to investigate a possible correlation between neural changes and clinical data.

**Discussion:**

In comparison to the non-acupoint, the results will firstly explore the superior effect between acupuncture with GB34 and EX-LE6 on GSD patients by observing their behavioral and neural response change to fatty-food cue, and then to investigate the underlying central mechanism.

**Trial registration:**

Chinese Clinical Trial Registry ChiCTR2000034368. Registered on 3 July 2020.

**Supplementary Information:**

The online version contains supplementary material available at 10.1186/s13063-021-05356-9.

## Background

Acupoint specificity is considered as one of the core scientific issues with respect to acupuncture practice [1]. According to the theory of Traditional Chinese Medicine (TCM), stimulation on acupoints elicits active responses that can have specific function for disease treatment [2]. In addition, the superior effect of acupuncture at acupoints on the disease-affected meridian (DAM) compared to those acupoints on the nonaffected meridian and sham ones has been demonstrated in several clinical studies [3–5]. It is speculated that the benefit of acupuncture on the DAM contributes to acupoint specificity. There are different kinds of acupoints with specific effect. For instance, the lower He-sea points are thought particularly effective for visceral disease treatment. Some extraordinary acupoints that belong to non-meridian points still have a therapeutic effect for visceral disease based on the millennium of clinical practice by ancient Chinese. However, differences among these acupoints and the underlying central mechanism still need to be further explored.

Acupuncture has been used to manage gastrointestinal and biliary pain and discomforts for thousands of years. Nowadays, it is gradually being accepted as an alternative and effective therapy in western countries for digestive disorders [6–8]. As one of the most prevalent gastrointestinal conditions and being associated with the highest socioeconomic costs [9–11], gallbladder stone disease (GSD) represents a significant public health problem in western countries, affecting 10–15% of adults [12]. In the USA alone, gallstones are present in 8 to 20% of the population by the age of 40 years [13, 14].

GSD is defined by the main occurrence of symptoms or complications caused by gallstones in the gallbladder and/or the bile ducts. Biliary colic (BC) is the main symptom of gallstone disease, and in addition, GSD patients often live with intolerance to fried or fatty foods (characterized by nausea, vomiting, and bloating) [15]. High-fat foods may strongly aggravate patients’ BC accompanying dyspeptic symptoms. Nowadays, the treatment algorithms for GSD remain predominantly invasive and based on surgery [16]. However, only about 60% of patients report the absence of abdominal pain after surgery [17]. In addition to that, the majority of them still have a limitation of fat in the diet, accompanying with some dyspeptic symptoms following the intake of fatty food, such as abdominal distension, nausea, and so on [18, 19]. These discomforts caused by high-fatty foods greatly compromised GSD patient’s quality of life.

Early in 2002, the World Health Organization reported that acupuncture is effective for BC [20]. Based on the meridian and viscera theory in traditional Chinese medicine, the acupoint of Yanglingquan (GB34), as the lower He-sea points of the gallbladder, has a specific effect for gallbladder disease. The extraordinary acupoints of Dannangxue (EX-LE6) are an experience point for treating gallbladder disease. Both the GB34 and EX-LE6 are frequently chosen in the clinic. And randomized controlled trials (RCTs) have shown that these two acupoints have satisfactory efficacy in relieving GSD patients’ discomforts [21–23].

Acupuncture is considered to have a regulating effect on the biliary system, including inhibiting the contractions of Oddi’s sphincter, bi-directionally regulating the gallbladder pressure, promoting the secretion of hepatic bile, and changing the content of the cholesterol in plasma and bile, and in further to reduce the formation rate of cholelithiasis, the number of gallstones, and the huge gallstones [24–28]. Recently, acupuncture on GB34 is found specifically activated the cognitive, motor, default network, and other parts of brain regions [29–32]. However, the underlying central effect of GB34 and EX-LE6 on the gallbladder of patients with GBD has not been covered.

In recent decades, the application of task fMRI makes it possible to provide evidence of human’s cerebral and behavioral response towards acupuncture stimulation [33–35]. Hence, in order to testify the hypothesis that either acupuncture with GB34 or EX-LE6 is superior in alleviating GSD patients’ symptoms compared to acupuncture with the non-acupoint, a fatty-food-cue task during MRI scan is designed to induce pain and accompanied discomforts of GSD patients and then the GSD patients’ behavioral and neural response change among all three groups before and after acupuncture will be compared. Therefore, the primary objective is to testify the therapeutic effect of acupuncture with GB34 and EX-LE6 in comparison to the non-acupoint and then further explore the superior effect between acupuncture with GB34 and EX-LE6 and finally investigate the potential central mechanism underlying acupuncture with GB34 and EX-LE6.

## Methods and design

### Study design

This is a parallel, randomized controlled task-fMRI study. Ninety participants will be recruited in this study, with 30 participants in each group.

**Fig. 1:**
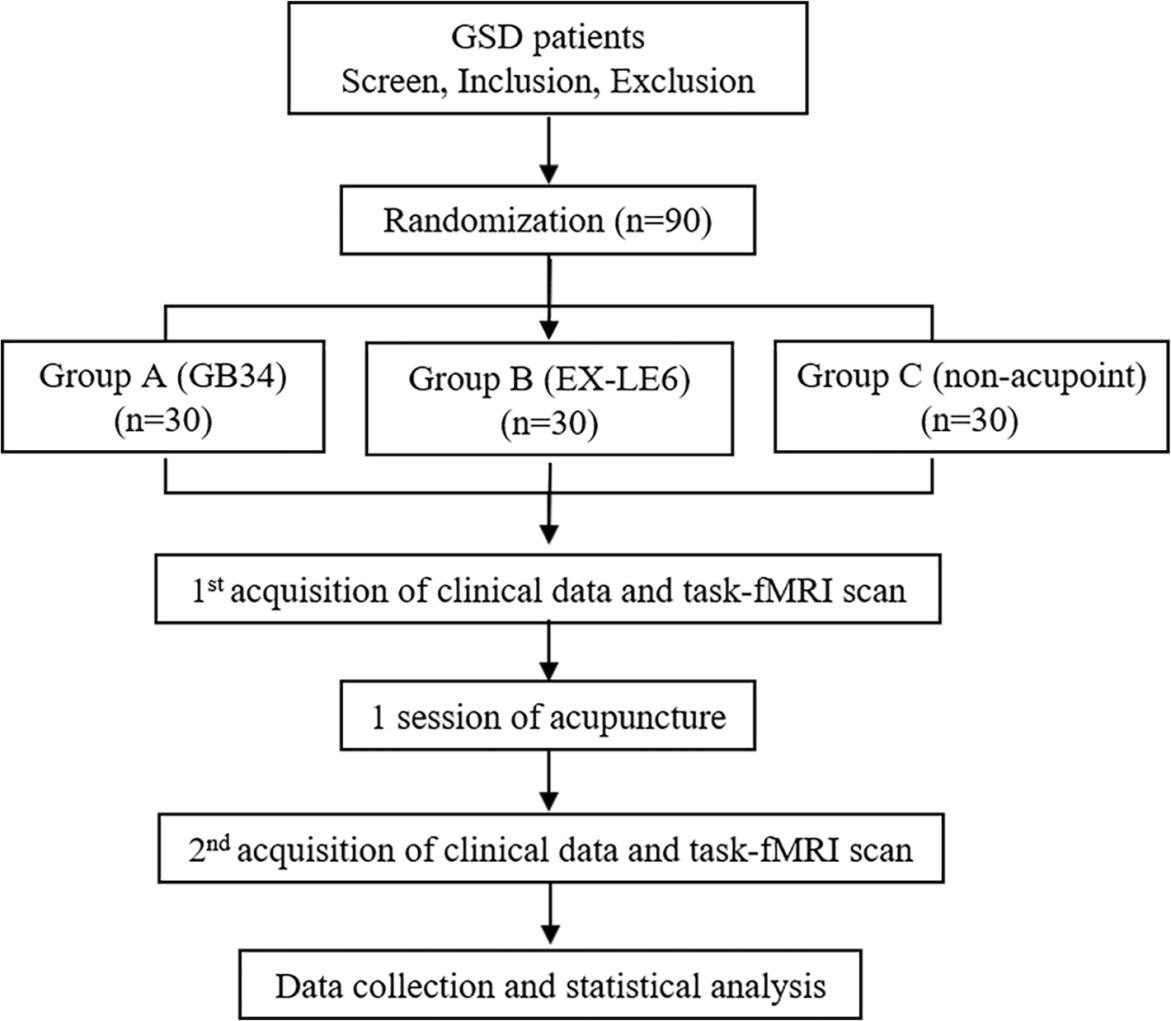
Study schedule: 90 eligible participants will be randomly allocated into three groups with a 1:1:1 ratio. Patients in each group will be randomly selected to undergo an fMRI scan. Imaging data and clinical data will be collected at baseline and at the end of the acupuncture treatment. GSD gallstone disease, fMRI functional magnetic resonance imaging

**Fig. 2:**
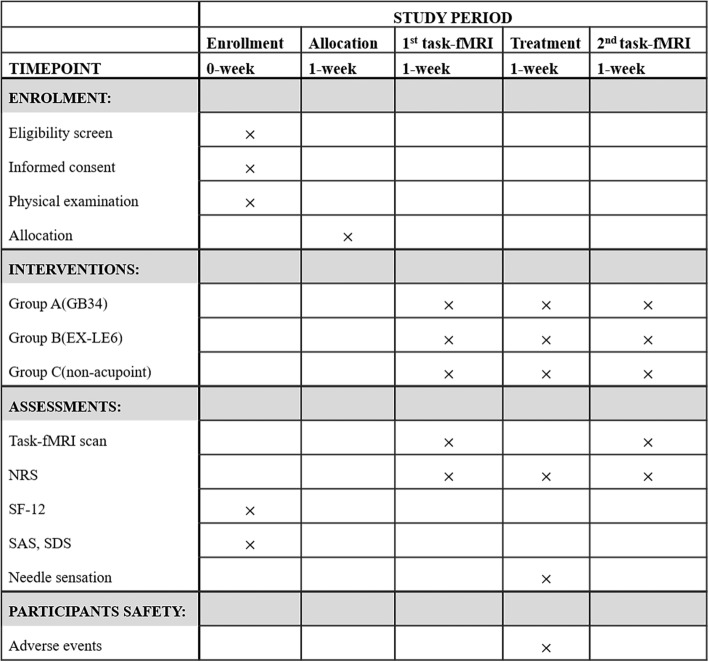
Standard Protocol Items: Recommendations for Interventional Trials (SPIRIT) schedule of the trial. This is a randomized controlled trial which includes a 2-day baseline period and one acupuncture treatment. In the baseline period, recruited patients will be screened according to the inclusion criteria and exclusion criteria; then, eligible GSD patients will give informed consent and receive a physical examination. After allocation, the patients will receive once GB34 acupuncture, EX-LE6 acupuncture, or non-acupoint. The outcome assessments and fMRI scans are performed at baseline and at the end of acupuncture. In addition, a physical examination will be performed at the baseline. Adverse events will be recorded in the CRFs at any time during treatment. fMRI functional magnetic resonance imaging, SF-12 12-item Short Form Health Survey, NRS Numeric Rating Scale, SAS Self-rating Anxiety Scale, SDS Self-rating Depression Scale

All participants in three groups will receive once 30-min immediate acupuncture stimulation. Clinical measurements and behavioral data of all participants during the task-fMRI scan will be assessed before and after the acupuncture treatment (Figs. 1 and 2). The protocol has been approved by the ethics committee of the 1st Teaching Hospital of Chengdu University of TCM (2019KL-029) and registered in the Chinese Clinical Trial Registry (NO: ChiCTR2000034368). This trial is reported in accordance with the Standard Protocol Items: Recommendations for Intervention Trials (SPIRIT) guidelines [36] (Additional file 1) and follows the principles of the CONSORT and STRICTA [37].

### Study setting and recruitment

The study will be carried out from August 2020 to December 2021 at the 1st Teaching Hospital of Chengdu University of TCM. The study will recruit participants with GSD, who meet the diagnostic criteria of the European Association for the Study of the Liver (EASL) [38] through outpatient and inpatient departments as well as social media platforms. Trained researchers will conduct participant screening.

### Participants

#### Inclusion criteria

(1) Age between 18 and 60 years and right-handed with normal or corrected-to-normal vision; (2) recurrent pain in the right upper abdomen over 3 months, accompanying with the aversion to high-fat food, nausea, bloating, etc.; (3) not taken any nonsteroidal anti-inflammatory drugs (NSAIDs), Chinese herbs, or other treatments for GSD within 2 weeks; (4) acupuncture naïve; and (5) signed informed consent.

#### Exclusion criteria

(1) Calculus in the bile duct; (2) acute cholecystitis, acute suppurative, gangrenous, incarcerated cholecystitis, and perforation of the gallbladder with diffuse peritonitis, etc.; (3) serious complications or primary diseases such as the cerebral, cardiovascular, liver, kidney, endocrine, and hematopoietic system; (4) severe digestive system diseases, such as peptic ulcer, upper gastrointestinal hemorrhage, gastric tumor, Crohn’s disease, irritable bowel syndrome, etc.; (5) white blood cells (WBCs) > 15 × 10^9^/L with systemic inflammatory response; (6) mental disorders such as serious anxiety and depression (the Zung Self-Rating Anxiety Scale (SAS) scores ≥70 or the Zung Self-Rating Depression Scale (SDS) scores ≥72); (7) having serious headache or migraine or having a history of head trauma or other central nervous system abnormalities; (8) being on weight-loss diets or taking other medications; (9) pregnancy or lactating women; (10) participating in any other clinical trials in the past 3 months; (11) received any acupuncture treatment in recent 1 month; (12) MRI contraindications, such as claustrophobia or implanted ferromagnetic metal.

### Drop out criteria

(1) Quitting themselves during research; (2) not following the requirements; (3) having serious illnesses, complications, or adverse events during research; (4) showing emotional instability or having drunk coffee, strong tea, or alcohol before fMRI scan.

### Informed consent and participant safety

If participants are eligible and interested in taking part in the study, they will be fully informed of the project information including the study procedures, benefits, and potential risks. Then, participants will be required to sign the informed consent to enter the study and have equal opportunities to receive proper treatment. Participants will be informed that they are free to withdraw from the study without penalty at any time.

To provide an overview of the health status of each participant, participants in this study would undergo a health evaluation including abdominal ultrasound, blood biochemical test, and electrocardiogram examination. During the acupuncture treatment, any adverse events such as pain, bleeding, fainting, or other severe events should be processed immediately and recorded in the case report forms (CRFs) carefully. In the food-cue task stage, any intolerable symptoms should be processed immediately and recorded in the CRFs carefully. In addition, we will follow up the patients twice within 1 month after the end of the experiment (once every 2 weeks) to ask whether they have any adverse reactions related to acupuncture or MRI.

### Randomization

Eligible GSD participants will be randomized in a ratio of 1:1:1 to group A (GB34), group B (EX-LE6), and group C (non-acupoint), with 30 patients in each group. Random sequence will be generated by a third-party using computer, placed in sealed opaque envelopes, and opened by an independent assistant in consecutive order.

### Blinding

Acupuncturists will not be blinded to treatment allocation for the different locations in each group. However, it is feasible to separate participants, outcome assessors, and statistical analysts. Participants will be told that there are three different types of acupuncture provided in this study. Participants in different groups will be separated into cubicles to refrain from communication. Outcome assessors and statistical analysts will be blind to the procedure and results of randomization, group allocation, and intervention.

### Sample size

According to our previous study, the Numeric Rating Scale (NRS) of GSD patients decreased by 2.4 points after acupuncture and by 0.85 points after non-acupoint acupuncture. In this study, we anticipate a reduction of NRS score by 2.4 points after acupuncture with GB34 or EX-LE6, and we anticipate a reduction by 0.85 points after acupuncture with non-acupoint. With α = 0.05, 1 β = 0.8, and a standard deviation of 2, we need at least 81 participants in total. Considering a 10% dropout, the sample size of 30 in each group, with a total of 90 participants, would be required in this trial. And 30 patients in each group have a stable statistical power for brain functional network analysis [39].

### Interventions

**Fig. 3:**
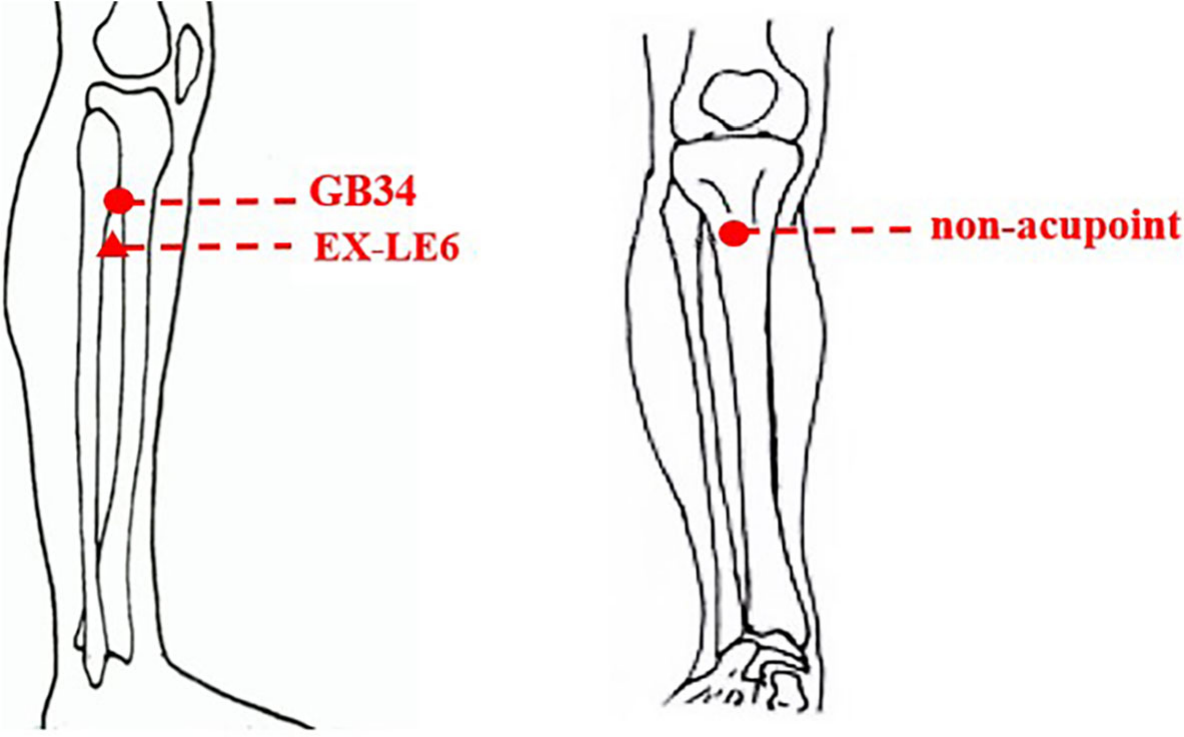
Locations of acupoints and non-acupoint. GB34 (Yanglingquan), in the depression anterior and inferior to the head of the fibula. EX-LE6 (Dannang), on the superior lateral aspect of the lower leg, 2 cun directly below the depression anterior and inferior to the head of the fibula. Non-acupoint, 1–2 cm outside of Zusanli (ST36)

Participants in all three groups received one acupuncture treatment. The acupoints used in the study include GB34 (used in group A) and EX-LE6 (used in group B). To explore the specific effects of acupoints of GB34 and EX-LE6 on GSD patients, a non-acupoint will be selected as the control. The non-acupoint (used in group C) is 1–2 cm lateral to Zusanli (ST36), which were selected in accordance with the findings of our previous studies [40] (Fig. 3).

Acupuncture treatment will be delivered to participants by one trained, licensed acupuncturist with at least 3 years of clinical experience. The acupuncturist will be trained and tested before participating in this trial.

The GB34, EX-LE6, or the non-acupoint will be punctured unilaterally (on patients’ right leg). Disposable sterile filiform needles (0.25 × 40 mm, Huatuo, Suzhou, China) will be perpendicularly inserted into acupoints at a depth of 21–26 mm after skin disinfection using alcohol; then, the needle is twisted between 90 and 180° and lifted and thrusted in an even amplitude between 0.3 and 0.5 cm, with 60 times to 90 times per minute to induce *deqi* sensation (including soreness, numbness, distention, and heaviness). After the *deqi* sensation is attained, needles will be retained for 30 min. During the 30 min, the above procedures will be manipulated for 10–15 s every 10 min to maintain the *deqi* sensation. This study aims to explore the superiority of the GB34 and EX-LE6 to the non-acupoint, so patients in group C will undergo an acupuncture procedure similar to the patients in the other two groups, but no deqi is required during the needle manipulation.

### Ethical consideration

During the acupuncture treatment, some other interventions, such as symptomatic relief or psychopharmacological medications, are usually not allowed to use. However, if participants experience discomforts that are difficult to tolerate, they would be treated immediately. Researchers should record all relevant information into the CRFs. Participants in group C can choose to receive acupuncture treatment or health consultation for free after completing this trial.

### Outcome measurements

#### MRI data acquisition

MRI data will be acquired with a 3.0-T magnetic resonance scanner (GE 3.0 T MR750, Wauwatosa, WI, USA) with an eight-channel phase-array head coil at the MRI Center in the University of Electronic Science and Technology of China.

Each subject will be scanned with a high-resolution structural image through a 3-dimensional T1-weighted sequence and blood-oxygen-level-dependent (BOLD) images with a Gradient-Recalled Echo-Planar Imaging (GRE-EPI) sequence. 3D anatomic image parameters are as follows: repetition time (TR) = 6 ms; echo time (TE) = 2 ms; flip angle = 12°; number of slices = 156; slice thickness = 1 mm; field of view (FOV) = 256 × 256mm^2^; data matrix = 256 × 256. BOLD-fMRI image parameters are 43 axial slices with a slice thickness of 3.2 mm, TR 2000 ms, TE 30 ms, flip angle 90°, FOV 220 × 220 mm2, and data matrix 64 × 64.

Before the MRI, subjects will be screened for contraindications and instructed to remove all magnetic and metal items. Foam padding is used to minimize head motion and earplugs dampened scanner noise. All subjects are in a supine position and keep awake during the entire scanning procedure.

#### fMRI paradigm

The fMRI paradigm used in this study involves two main conditions, which are viewing of high-fat food pictures and low-fat food pictures. It is meant to investigate the immediate effect of acupuncture on the brain response to fatty food and its association with patients’ behavior.

#### Food (picture)-cue task

**Fig. 4:**
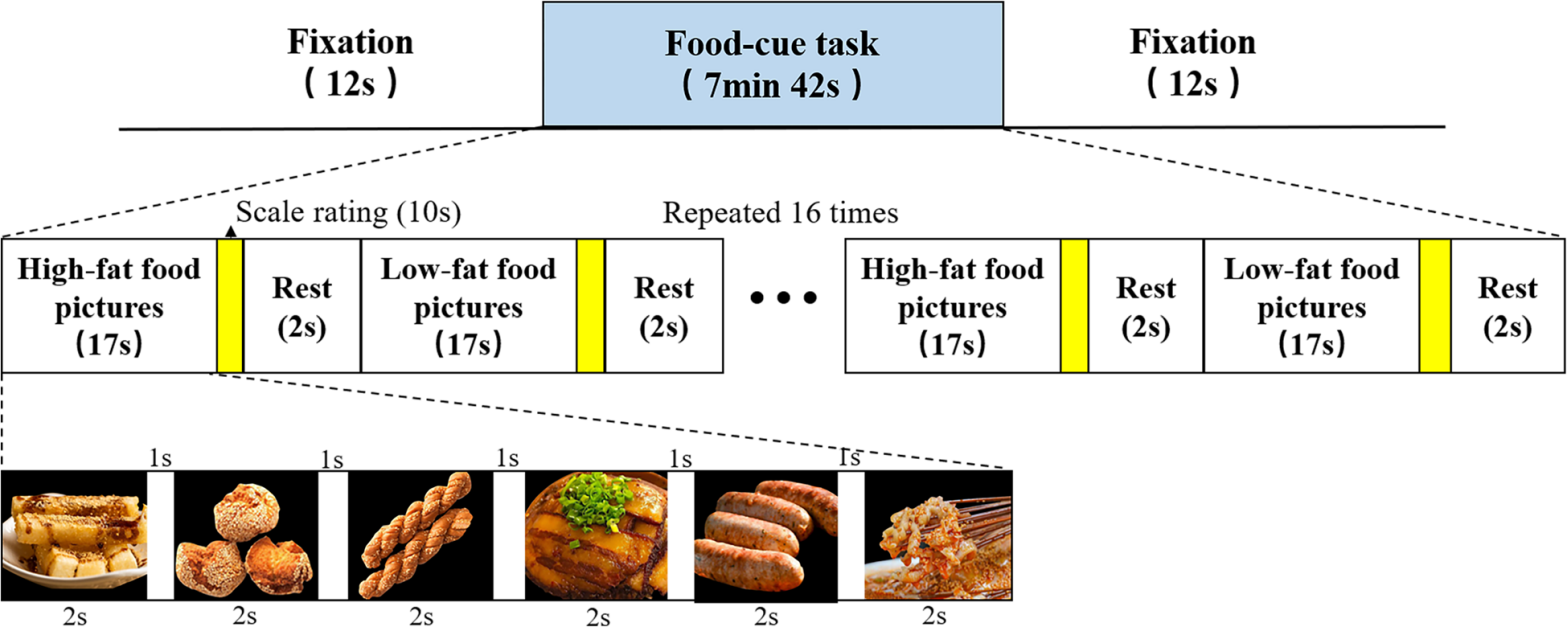
The food picture stimulation paradigm during fMRI. The food-cue fMRI task runs 7 min 42 s for each participant. In the beginning of the paradigm, a 12-s pre-fixation “+” appears in the center of the screen to keep the participant’s attention. After the task is completed, a post-fixation “+” appears for 12 s to end the task. A total of 16 blocks (8 high- and 8 low-fat food blocks) will be programmed. In each block, six cue high-fat food pictures or low-fat food pictures will be presented pseudo-randomly. Each picture lasts for 2 s with 1-s interval. Each block costs 17 s, with 12-s interval to the next block. In the end of each block, subjects will be asked to rate on an 11-point Likert scale for 10s. When the rating is completed, the screen will become blank for 2 s and then a new block would begin to appear

Participants will complete the food (picture)-cue task in the MRI scanner twice: before and after 30-min acupuncture treatment. The visual food-cue task, previously validated for fMRI [41, 42], was performed with a block design (Fig. 4).

Specifically, a total of 16 blocks (8 high- and 8 low-fat food blocks) will be programmed using the E-prime 3.0 software (Psychology Software Tools, Pittsburgh, PA, USA). In each block, six cue high-fat food pictures (e.g., soy-braised pork, cream cake) or low-fat food pictures (e.g., apple, celery) will be presented pseudo-randomly. Each picture lasts for 2 s with 1-s interval. Each block costs 17 s, with 12-s interval to the next block. In the end of each block, subjects will be asked to rate “How strong is your desire to eat your favorite food shown before?” (range from −5 highly aversive to + 5 highly appealing) on an 11-point Likert scale for 10s, by pressing two buttons with their right index and middle fingers [43]. When the rating is completed, the screen will become blank for 2 s and then a new block would begin to appear. In the beginning of the paradigm, a 12-s pre-fixation “+” appears in the center of the screen to keep the participant’s attention. After the task is completed, a post-fixation “+” appears for 12 s to end the task. The food-cue fMRI task runs 7.7 min (462 s) for each participant. Participants lie supine in the scanner and viewed a screen through a mirror.

#### Pictures selected for the food-cue task

A total of 96 pictures are from various websites such as Burst (a free stock photo platform, https://burst.shopify.com). Forty-eight sweet/savory high-fat food pictures and 48 sweet/savory low-fat food pictures are selected. Pictures are processed using Photoshop 7.0 (Adobe Systems, San Jose, CA), and the parameters are 800 × 600 resolution, 300 dpi, 4 × 3 aspect ratio [44].

An online questionnaire will be created and released by social media (https://www.wjx.cn) and 20 healthy subjects and 20 patients with gallstones outside the study would complete it. The questionnaire aimed to verify if the Chinese food pictures could be accurately recognized and habitually consumed and that they are correctly perceived in terms of the content of fat, calorie, and sweet/savory taste. The food pictures will be used in experiments only if they are considered adequate [45]. The specific evaluation process is presented in an additional file (Additional file 2).

### Primary outcome measurement

The primary outcome will be measured by the NRS. NRS is a commonly used, validated, and reliable measure of the current self-reported pain intensity [46, 47]. NRS is a horizontal line with 10 segments, being labeled from 0 (no pain at all) to 10 (the worst pain imaginable). The NRS will be assessed at four timepoints: before and after the 1st task fMRI scan (before acupuncture), after acupuncture (before the 2nd task fMRI scan), and after the 2nd task fMRI scan.

### Secondary outcome measurement

The needle sensation evaluation form will be asked to fill after receiving one acupuncture treatment. The form is based on the Chinese version of the modified Massachusetts General Hospital Acupuncture Sensation Scale (C-MASS) and its validity and reliability have been examined [48, 49].

### Data management and monitoring

For this trial, relevant data, adverse events, and safety assessments will be documented in both paper and electronic versions of CRFs. Each participant has a unique numeric identifier. Only outcome assessors have access to CRFs and do the double data entry. The Evidence-based Medicine Center of the Chengdu University of TCM will be in charge of supervising the study and monitoring data every 3 months.

### Statistical analysis

#### Clinical data analysis

The clinical data will be statistically analyzed by SPSS 22.0 software (IBM SPSS Statistics, IBM Corp, Somers, New York). Skewed data distribution will be normalized. Means, standard deviations, and 95% confidence intervals (CIs) will be computed to describe continuous variables. Since only one session of acupuncture will be conducted in our study, the per-protocol (PP) analysis will be used, and the subjects with missing data will be excluded. The reasons for missing data should be recorded in detail.

#### Clinical outcome analysis

Comparison of NRS scores between four timepoints among three groups will be made using a repeated-measures ANOVA. Further, the planned contrast of changed NRS scores (before vs. after acupuncture; before vs. after fatty-food cues) will be conducted with a one-sided exact test for which *p* < 0.05 is considered significant.

#### In-scanner behavioral rating analysis

For the 11-Likert scale scores obtained during the task, we employ a 3 × 2 × 2 repeated-measures ANOVA with the group (group A, group B, group C), timepoint (pre-acupuncture, post-acupuncture), and condition (high-fatty food pictures, low-fatty food pictures). Post hoc analyses will be conducted using Tukey post hoc or Bonferroni comparisons. And the post hoc significant differences (p < 0.05) will be followed up with t-tests. The data will be considered statistically significant with a level of 0.05.

#### Neuroimaging data analysis

All image preprocessing and general linear modeling will be done using SPM12 software (SPM12, Wellcome Department of Imaging Neuroscience, London, UK; www.fil.ion.ucl.ac.uk/spm/) performing on MATLAB 8.6 (Mathworks, Inc., Natick, MA, USA). Data preprocessing will include the first 5 timepoints discarding for magnetic saturation effects, slice timing, head-motion correction, spatial normalization in Montreal Neurological Institute (MNI) space resampled at 3 mm × 3 mm × 3 mm voxel size, filtering, and spatial smoothing using an 8-mm FWHM Gaussian kernel.

After preprocessing, the general linear model (GLM) will be used to model task-related effects. The inter-block interval serves as an implicit baseline. The two task conditions (high- and low-fat food), the 10-s 11-Likert scale rating period, and six head-motion parameters to control motion-related artifacts will be designed for the first-level design matrix. The following contrast (high-fat food cues > low-fat food cues), for each subject and for each session (before and after acupuncture treatment), will be then used for the second-level group analysis.

Furthermore, for correlation analyses, a Spearman correlation analysis will be performed to investigate a possible correlation between neural changes and clinical data.

## Discussion

To explore the superior effect between acupuncture with GB34 and EX-LE6 on reliving GSD patients’ behavioral and neural response and the underlying central mechanism, the RCT is designed with a fatty-food-cue task to induce patients’ behavioral and neural reactivity. To our knowledge, this will be the first parallel, randomized study of acupuncture in combination with clinical questionnaire evaluations and task-fMRI among digestive diseases. The results are based on the NRS outcome and objective outcomes from the fMRI study, which may help provide more convincing evidence that immediate acupuncture is effective for alleviating pain and accompanied symptoms such as aversion to fatty food, vomiting, nausea, etc. for GSD patients.

The close relationship between acupoints and viscera is the basis of the bi-directional therapeutic effect of acupuncture for viscera disease [50]. In the theory of traditional Chinese medicine, the selection of the acupoint is crucial to the curative effect. Although many studies have demonstrated verum acupoint superior to non-acupoint in alleviating pain and suffering [51, 52], the acupoint specificity remains controversial. GB34 locates in the gallbladder meridian, which is particularly effective for gallbladder disease treatment. Moreover, it has been concluded that the hypothalamus-limbic system was significantly modulated by acupuncture at GB34 rather than at non-acupoint [53]. Hence, in comparison to the non-acupoint, this trial aims to firstly explore the superior effect between acupuncture with GB34 and EX-LE6 on GSD patients by observing their behavioral and neural response change to fatty-food cue and then to investigate the underlying central mechanism. This may have important implications for the effect of acupuncture in improving biliary and gastrointestinal symptoms.

On the condition of deficient motility of the biliary system with stones stuck in the gallbladder and cystic duct, bile acid transmission is blocked and the normal digestion of fat in the gastrointestinal system is affected [54]. Acupuncture, especially on the acupoint GB34, has been demonstrated not only for biliary colic relief, but also for having regulating effect on the biliary system [24–28]. However, the underlying central mechanism has never been investigated to date. Therefore, a fatty-food-cue task during an MRI scan is designed to induce pain and accompanied discomforts of GSD patients to investigate the immediate effect of acupuncture on the brain response and its association with patients’ behavior.

To improve the reliability and repeatability of study results, a strict quality control is indispensable. In this study, quality control will be strengthened focus on baseline consistency, strict task design, and fMRI scan, as well as the trained acupuncture manipulation. Participants will be screened strictly according to inclusion and exclusion criteria. The selected food pictures are edited with a unified standard to ensure the identification and visual homogeneity. And prior to the food-cue task, these pictures would be evaluated using online questionnaires. All scans are performed by the same scanner and operator. The same experienced acupuncturists with standard operation procedure are in charge of acupuncture treatment. There are still some limitations that should be noted in this study. Firstly, the one-time acupuncture treatment may be insufficient to achieve a measurable response. Prolonged treatment periods should be considered in the future study. Secondly, as the unique nature of acupuncture and many patients in China are familiar with *deqi* sensation, it is impossible to completely blind all participants. Another limitation is that the primary and secondary outcome measures in this study are self-rating scale, where the outcome assessors may not be able to blind to group allocation and intervention.

In conclusion, the results of this study will allow to observe the superior effect between acupuncture with GB34 and EX-LE6 on alleviating GSD patients’ symptoms. In addition, the potential central mechanism underlying acupuncture with GB34 and EX-LE6 will be further investigated.

## Trial status

This trial was registered on 3 July 2020 (registration number: ChiCTR2000034432; version number: 2.1; version date: 1 January 2021). This trial is still recruiting patients now. This trial is currently in the stage of recruiting patients. The first participant was included on 26 August 2020. To date, 6 participants have been recruited. And the trial is expected to end on 10 December 2021.

## Supplementary Information

**Additional file 1.** Standard Protocol Items: Recommendations for Intervention Trials (SPIRIT) guidelines.

**Additional file 2.** Pictures selected for the food-cue task.

## Data Availability

Not applicable. This is only a study protocol and as such no unpublished data are available.

## Abbreviations

GSD: Gallbladder stone disease
BC: Biliary colic
CCK: Cholecystokinin
GB34: Yanglingquan
EX-LE6: Dannang
fMRI: Functional magnetic resonance imaging
RCTs: Randomized controlled trials
SPIRIT: The Standard Protocol Items: Recommendations for Intervention Trials
EASL: The European Association for the Study of the Liver
NSAIDs: Nonsteroidal anti-inflammatory drugs
WBCs: White blood cells
SAS: The Zung Self-Rating Anxiety Scale
SDS: The Zung Self-Rating Depression Scale
CRFs: Case report forms
ST34: Zusanli
TR: Repetition time
TE: Echo time
FOV: Field of view
BOLD: Blood-oxygen-level dependent
GRE-EPI: Gradient-Recalled Echo-Planar Imaging
NRS: The Numeric Rating Scale
SF-12: The 12-item Short Form Health Survey
SF-36: The 36-item Short Form Survey
C-MASS: Chinese version of modified Massachusetts General Hospital Acupuncture Sensation Scale
CIs: Confidence intervals
ANOVA: One-way analysis of variance
MNI: Montreal Neurological Institute
GLM: General linear model
