# Supplementary material for: The cerebral mechanism underlying the acupoints with specific effect for gallbladder stone disease: protocol for a randomized controlled task-fMRI trial
Source: Trials. 2021 Jun 14;22:399. doi: 10.1186/s13063-021-05356-9 (PMC8204415; doi:10.1186/s13063-021-05356-9)
Supplement: Supplementary file 2 — Additional file 2. Pictures selected for the food-cue task. [file 13063_2021_5356_MOESM2_ESM.docx]

| 1. Each food picture was presented one at a time and the respondents were asked the following questions | |
| --- | --- |
| **Questions** | **Answer** |
| **Do you recognize this food?** | - **yes;** - **I am not sure;** - **no** |
| **Have you already eaten this food?** | - **yes;** - **I am not sure;** - **no** |
| **Do you like this food?** | - **yes;** - **more or less;** - **no** |
| **2. Participants were asked to rate on a 7-point Likert scale (1=‘almost nothing’ ; 7=‘extremely’)** | |
| **How much fat does this food contain?** | **Rate on the range of 1 to 7** |
| **How sweet is this food?** | **Rate on the range of 1 to 7** |
| **How calorie-dense is this food?** | **Rate on the range of 1 to 7** |

**Additional file 2. Pictures selected for the food-cue task.**

Pictures valued questionnaire.

Pictures that show kinds of Chinese cuisines will be valued by this questionnaire. Food pictures were considered adequate if:

1) 80% of the sample recognize, habitually consume them, and like them;

2) and if their mean values were above 5 for the high content of fat, calorie and sweet/ savory taste;

3) and if their mean values were lower than 3 for the low content of fat, calorie and sweet/savory taste.
